# Nutritional status, nutrient imbalances, food-related behaviors and dietary supplements use among patients with celiac disease in Lebanon: a national cross-sectional study

**DOI:** 10.12688/f1000research.121859.1

**Published:** 2022-07-01

**Authors:** Maha Hoteit, Zeinab Chamas, Shaza Assaf, Malek Michael Bouhairie, Abbas Bahr, Romy Daccache, Rami Matar, Marwa Hallal, Michael Maitar, Mahmoud Hallal, Samer Hotayt, Bilal Hotayt

**Affiliations:** 1 Faculty of Public Health, Lebanese university, Beirut, Lebanon; 2 PHENOL Research Group (Public HEalth Nutrition prOgram Lebanon), Faculty of Public Health, Lebanese University, Beirut, Lebanon; 3 Lebanese University Nutrition Surveillance Center (LUNSC), Lebanese Food Drugs and Chemical Administrations, Lebanese University, Beirut, Lebanon; 4 Gastroenterology Department, Faculty of Medical Science, Lebanese University, Beirut, Lebanon; 5 Gastroenterology Department, Bahman hospital, Beirut, Lebanon; 6 Faculty of Medicine, Lebanese American University, Byblos, Lebanon; 7 Saint George’s University, Grenada, USA; 8 Department of Medicine, Division gastroenterology and hepatology, Southern Illinois University, Springfield, USA; 9 Anesthesia department, Saint Joseph Hospital, Paris, France; 10 Gastroenterology Department, Sahel General Hospital, Beirut, Lebanon

**Keywords:** Celiac disease, gluten free diet, anthropometry, biochemical, Lebanon

## Abstract

**Background**: Celiac disease (CD) is an autoimmune disorder triggered by gluten, that occurs in susceptible individuals and is associated with dietary restriction and subsequent nutritional deficiencies. This study investigated the diet quality, nutrition imbalances and nutrition status among young children, adolescents and adults with CD who were referred to several hospitals in Lebanon.

**Methods:** A cross-sectional study in 50 individuals (31.74 ± 15.64 years) with CD was conducted, using biochemical parameters, anthropometric measurements, dietary and physical activity assessments.

**Results**: Of the 50 participants, 38% and 16% were presenting low serum levels of iron and vitamin B12, respectively. The majority of participants were physically inactive and around 40% of them had low muscle mass. A weight loss of 10% to 30% indicating mild to moderate malnutrition was shown in 14% of individuals. The assessment of food-related behaviors shows that 80% of participants were reading nutrition labels and 96% of them were following gluten-free diets (GFD). Some barriers including family ignorance (6%), language of the nutrition labels (20%) and expensive GF products (78%) were limiting the adherence to GFD. The inadequacy of the daily energy intake along with insufficient intakes of calcium and vitamin D were remarked among individuals with CD. However, protein and iron intake were exceeding the recommendations among all age groups, except in males aged 4-8 years and 19-30 years. Half the study participants were using dietary supplements where 38%, 10%, 46%, 18%, 16% and 4% used vitamin D, vitamin B12, iron, calcium, folate and probiotics, respectively.

**Conclusion:** GFD is the key treatment for CD. However, it is not without inadequacies and may cause certain deficiencies such as calcium and vitamin D leading to reduced bone density. This underlines the critical role of dietitians in education and maintenance of healthy GFD among individuals with CD.

## Introduction

Celiac disease (CD) is a chronic inflammatory autoimmune disorder characterized by villous atrophy of the small bowel. ^1^ The exposure to dietary wheat gluten and proteins of rye and barley triggers CD among genetically predisposed persons. ^2^ In certain circumstances, individuals show no symptoms and the diagnosis is only possible through serologic screening. ^3^ According to the European Society for the Study of Celiac Disease (ESsCD) guideline, the diagnostic criteria depend upon the exposure to dietary gluten or related proteins of rye and barley-dependent symptoms, through some histopathologic findings (villous atrophy and crypt hyperplasia) from a biopsy of the duodenum CD, specific antibody levels, HLA-DQ2 and/or HLA-DQ8. ^4^ The prevalence of CD is estimated to be one to two individuals per 100 worldwide ^5^ and roughly one in 99 among children and adolescents. ^6^ Moreover, according to a recent systematic review and meta-analysis, the pooled seroprevalence of CD in the general population is significantly higher in children (2%) compared with adults (1%, p-value=0.01) and significantly greater among females (1.65%) compared to males (0.8%, p-value=0.04). ^7^ The pooled sero-prevalence of CD in the Middle Eastern area (1.47%) is higher than that in South Asia (1.25%) and East-Asia (0.06%). ^7^ The highest prevalence among the Arab regions was reported in Saudi Arabia (3.2%), and the lowest in Tunisia (0.1%). ^8^ In Lebanon, the prevalence of CD was around 0.5% in 2011. ^9^ Furthers, women have shown elevated prevalence compared to men in the Arab countries ^8^ and according to solid evidence, the risk becomes common and high if a first- or second-degree relative has been previously diagnosed. ^6^

CD affects the proximal part of the small intestine, which in turn results in micronutrient malabsorption, particularly iron, folic acid, vitamin B12, calcium and vitamin D, resulting in anemia on the short term and reduced bone density on the long-term. ^2^ Further, beyond inflammation and malabsorption, CD is characterized by recurrent abdominal pain, nausea, vomiting, steatorrhea and loss of weight which also compromise the nutrition status of affected individuals. ^6^ Until today the only therapy for individuals with CD remains the adherence to a gluten-free diet (GFD) along with the avoidance of foods containing wheat, rye, and barley derivatives. ^10^ According to several studies, the adherence to a strict GFD can reduce the risk of health-related complications, for instance: malignancy, osteoporosis, growth retardation in children, liver disorders and reproductive tract disorders. ^1^ According to recent data, the adherence to GFD has remained unchanged over the past twenty years ^11^^,^^12^ and a considerable number of individuals with CD do not adhere to the dietary restrictions, which affects the patient’s quality of life and causes compromised nutrition status. ^10^ Moreover, some individuals with CD may look for other approaches to treatments such as complementary and alternative medicine (CAM) which include dietary supplements (DS) use. ^13^ There is scarce data on the DS use in CD. Many studies identified the DS use among CD patients to be within a range of 21.6% to 23.6%. ^13^ This percentage was less than the percentage of DS use among the general population (27%). ^13^ Thus, investigating the nutrition status through dietary assessment of individuals with CD is warranted especially among children, adolescents, and adults to avoid malnutrition and related morbidity. Such an evaluation of GFD adherence should be executed by experienced trained dietitians; however, the access to such dietitians with expertise in the field of CD may be limited. Beyond GFD adherence, many reasons exist for the necessity of nutritional investigation including the poor public awareness about CD, and the lack of regulations for the production and analysis of gluten-free labelled products. ^14^ To the best of our knowledge, there are no published data that address the nutrition situation and dietary supplements use among individuals with CD in Lebanon. Therefore, the aim of the present study was to assess the nutritional status, food-related behaviors, caloric, macro- and micronutrients intake and dietary supplements use among individuals with CD in Lebanon.

## Methods

### Study design and participants

*Selection procedure*

Participants from all age categories were invited to participate in the current cross-sectional study between February and July 2018. A random selection was performed. Participation in the study was proposed to all patients who came to the pediatrician-gastroenterologists and gastroenterologists in three hospitals in Beirut and Mount Lebanon for a follow-up visit and met the criteria for inclusion, which was a diagnosis of CD based on serological and histological markers according to the international guidelines, ^15^ more than six months prior to study enrolment to guarantee adequate GFD knowledge. All participants with no proven biopsy, pregnant women, history of cancer, eating disorders, malnutrition, renal diseases and any other factors that affect the nutrition situation, were excluded.

*Questionnaires and dietitians’ assessment*

The survey contained 78 questions concerning demographics, disease’s characteristics, history and presentation, food-related behaviors, lifestyle and diet history, weight before and after being diagnosed with CD, adherence to GFD and social and emotional barriers for sustaining the GFD. The questionnaire was derived from the one available from the Canadian Celiac Association website and used previously in the “Canadian Celiac Health Survey”. ^16^ Medical history was evaluated based on the age at diagnosis of CD, family history of CD, symptoms (diarrhea, headache, flatulence and bloating), comorbid diseases (food allergies, food intolerance, other non-communicable disease). Alongside the first questionnaire, additional FFQ for caloric and protein intake, calcium, vitamin D and iron intake was addressed to individuals with CD along with questions on dietary supplement use. This FFQ was derived from two Lebanese-validated questionnaires to address the questions for children, adolescents and adults ^17^^,^^18^ in which a substitution of 24 foods with GF foods was executed (see *Underlying data* ^69^). Dietary supplement use was assessed through patient’s response of “Yes” or “No” to the statement: “I use dietary supplements in addition to GFD”. To investigate the type of supplements used, patients were asked to answer the following question: “Since you were diagnosed, have you used any of the following dietary supplements?” The specific options included vitamin D, probiotics, iron, folate, calcium, and vitamin B12, which are the commonly used DS among individuals with CD. ^19^ The questionnaire used in the present study was reviewed by two national and one international expert in CD and was pre-tested in 14 non-expert individuals as a focus group. The aim of the focus group was to explore a specific set of issues: the questionnaire’s readability, reliability and inter consistency (Cronbach-alpha=0.69). Moderators often commence the focus group by asking broad questions about the topic of interest, before asking the focal questions. Although participants individually answer the facilitator's questions, they are encouraged to talk and interact with each other. In addition, three-day food records were collected and analyzed using Nutritionist Pro software (version 3.2, AXXYA) to assess caloric, micro- and macronutrients intake. The mean value and the standard deviation (SD) were used when interpreting the nutrient’s intake.

Weight and height were measured using automated scale and stadiometer. Body composition including body fat, lean body mass and visceral fat were assessed using Bio-impedancemetry body composition analyzer (BF-511, OMRON). The standard formula of body mass index (BMI) was calculated by dividing weight in kilograms (kg) by height in meters squared (m ^2^).

*Laboratory values*

According to a study by Bledsoe *et al.* (2019), ^19^ and Marteau *et al.* (2001) ^20^ albumin and micronutrient deficiencies, including vitamins B12 and D, as well as folate, iron, zinc and copper, are common in adults at the time of diagnosis with CD. Thus, the study participants were tested for three out of six micronutrients (serum iron, folate and vitamin B12) along with albumin levels in the present study. Venous blood was collected after a 12-hour period of fasting, centrifuged and stored at −80°C. The iron level was measured in serum by the *in vitro* assay method with Roche Diagnostic (Mannheim, Germany) and Hitachi 704 devices (Roche Diagnostics, Switzerland).

All patients underwent a complete hemogram. However, only albumin, serum iron, folate and vitamin B12 were reported in this study to comply with the outcomes discussed in Bledsoe *et al.* ^19^ Serum iron was diagnosed according to the criteria followed by the laboratory in which the analysis occurred. ^21^ The reference values for children were: 8.9-21.4 μmol/L (SI units), males (adults): 14-32 μmol/L (SI units) and females (adults): 11-29 μmol/L (SI units) were compared to the values obtained in the current study. Serum folate and vitamin B12 levels were measured using a competitive immunoassay. As for vitamin B12, the reference values for 1–<11 years: 260–1200 pmol/L, 12–<18 years: 200–800 pmol/L, 18–<65 years: 200–600 pmol/L ^22^ were also used to compare the results obtained in this study. Concerning the folate reference values, the normal serum levels were, for children: 5-21 ng/ml and for adults: 2-20 ng/mL. ^23^ As for albumin, it was considered low if the value was <3.5 and high if >5.5 g/dl. ^24^

*Food-related behaviors and GFD dietary adherence*

Some food patterns including shopping, cooking and eating out habits were assessed among the study participants. The adherence to GFD was assessed by trained dietitians who investigated the infringement regarding dietary recommendations. ^12^ Across the three-day food records, patients presenting a consumption between 3 to 10 g of gluten per day were considered as non-adherent. ^12^^,^^25^

*Facilitators and barriers*

To search for possible barriers, patients were asked, “what are the major challenges that limit the adherence to a strict gluten-free diet?”. Trained dietitians asked participants to report whatever came to mind concerning this question. The answers were analyzed according to the methodology used by Braun and Clarke. ^26^

*Indicators of malnutrition and criteria for the determination of nutritional status*

The determination of the nutritional status was based on anthropometric data (weight, height, BMI), hypoalbuminemia and the percentage of weight loss taking into consideration age and sex factors. A weight loss of 10% to 20% indicated mild malnutrition, a weight loss of 20% to 30% characterized moderate malnutrition, and a weight loss of more than 30% indicates severe malnutrition. ^25^

*Physical activity*

Several types of physical activity such as aerobic (fitness), swimming, running, strength training gym, walking, cycling and yoga were investigated. The frequency of physical activity was based on frequency per week: one time per week, two-three times per week, four or more per week, daily or no physical activity.

### Statistical analysis

Statistical analysis of the study was performed using SPSS 28.0 (IBM Statistical Package for Social Sciences software version 28). The descriptive data were presented as percentages for categorical variables and mean ± SD for quantitative variables. Categorical variables were compared with the Chi-square test and continuous variables were compared with the student-T test. P<0.05 was considered a statistically significant difference with a 95% confidence interval.

### Ethical considerations

At the beginning of the study, a consent form was signed by participants. Moreover, this study received the approval of the ethics committees at the Lebanese university (CU#18-46). In addition, an assent form was signed by the parents of children and adolescents under 18 years old.

## Results

A total of 50 participants (78% females; mean age=31.74 ± 15.64 years) was enrolled in the present study. Out of 50, eight participants were aged below 18 years. Age categories were classified according to the dietary reference intake classes ^27^ of which 46% of the population’s age ranged between 31 and 50 years. In the current study, females were younger than males (p-value=0.03). Moreover, around 66% of all the participants had university degrees and more than 70% were remunerated for more than 1,500 million Lebanese pounds (LBP) per months. Half the study’s participants were married. Overall, in almost all the characteristics, there were no significant differences between females and males in this study (p>0.05). The sociodemographic characteristics of the study participants are displayed in Table 1.

**Table 1.** Socio-demographic characteristics of biopsy-proven study population, overall and by gender. LBP: Lebanese Pounds.

Variables	Overall (N=50) mean (SD)/N (%)	F (N=39;78%) mean (SD)/N (%)	M (N=11; 22%) mean (SD)/N (%)	p-value
Age (years) Mean±SD ^a^	31.74±15.64	34.15±2.50	23.18±3.58	0.03
Age groups ^b^				
4-8	5.5±1.64/6(12)	6.0±0.91/4(10.5)	4.5±0.5/2(18.2)	0.37
9-13	12.6±1.15/2(4)	13±1.0/1(2.6)	12±0.0/1(9.1)	0.66
14-18	-	-	-	-
19-30	24.5±3.34/12(24)	25±1.17/7(18.4)	23±1.52/5(45.5)	0.36
31-50	38.08±5.6/23(46)	38.3±1.59/20(52.6)	37.6±1.67/3(27.3)	0.78
51-70	57.5±5.2/7(14)	58.25±3.19/6(15.8)	56±2.0/0(0)	0.67
Income level per month (%)				0.47
≥1,500,000 LBP	36 (72)	29 (74.4)	7 (63.6)	
<1,500,000 LBP	14 (28)	10 (25.6)	4 (36.4)	
Education level (%)				0.64
Illiterate	0 (0)	1 (2.6)	0 (0)	
School	11 (22)	9 (23.1)	2 (18.2)	
High school	6 (12)	5 (12.8)	1 (9.1)	
University	33 (66)	24 (61.5)	8 (72.7)	
Marital status (%)				0.6
Single	23 (46)	17 (43.6)	6 (54.5)	
Married	26 (52)	21 (53.8)	5 (45.5)	
Divorced	1 (2)	1 (2.6)	0	

^a^ SD=standard deviation.

^b^ classification according to the DRIs values. ^27^

Table 2 includes the medical history, anthropometric measures and lifestyle characteristics of the study participants. In the current study, males were taller than females (p-value=0.042). It was observed that the weight of all participants increased in a range of 4 to 5.6 kg since being diagnosed with CD. This was significantly higher among males compared to females (p-value=0.023). Moreover, 45% of females were overweight compared to 37% males. The assessment of malnutrition was based on Kotze *et al.* ^25^; a weight loss of 10% to 30% indicating mild to moderate malnutrition were shown in 14% of the study population. The body composition analysis conducted for the study participants revealed that 40% of them had low muscle mass (5.0 to 32.8% for males and 5.0 to 25.8% for females). ^28^ Further, the mean muscle mass was higher in males compared to females (p-value=0.034). In total, 60% of participants were having high fat mass (more than 20% in males and more than 30% in females). In addition, the mean value of fat mass among women where higher than that among men (p-value=0.014). Furthermore, the majority of the study participants had low visceral fat and there were no significant differences regarding the mean and SD of visceral fat among both genders (p-value=0.25).

**Table 2.** Anthropometry measures, medical and lifestyle characteristics of the study population, overall and by gender. CD: celiac disease; BMI: body mass index.

Variables	Overall (N=50) mean (SD)/N (%)	F (N=39;78%) mean (SD)/N (%)	M (N=11; 22%) mean (SD)/N (%)	p-value ^d^
**Anthropometry indices**				
Weight (kg)				
Weight before GFD ^a^	59.99±2.69	41.54±6.22	57.92±4.97	**0.025**
Current weight	64.31±3.27	44.02±7.06	63.60±5.06	**0.017**
Weight change ^b^	4.32±1.84	3.93±2.29	5.68±2.03	**0.023**
Risk of malnutrition (weight loss in the past 6 months)				0.31
No risk (<10%)	43 (86)	32 (82.1)	11 (100)	
Mild (10%-20%)	6 (12)	6 (15.4)	0 (0)	
Moderate (20%-30%)	1 (2)	1 (2.6)	0 (0)	
High risk (>30%)	0 (0)	0 (0)	0 (0)	
Height (cm)	159.56±18.62	145.45± 7.04	161.9±8.23	**0.042**
BMI adults ^c^ (≥19 years)				
<18 kg/m ^2^	2 (5)	2 (6.1)	0 (0)	0.66
18-25 kg/m ^2^	21 (52)	16 (48.5)	5 (62.5)	
≥25 kg/m ^2^	18 (43)	15 (45.5)	3 (37.5)	
Muscle mass (kg) (≥6 years) ^28^	27.31±7.03	26.21±0.72	31.47±3.9	**0.034**
Low	20 (40)	15 (39.5)	5 (50)	
Normal	23 (46)	23 (60.5)	0 (0)	
High	1 (2)	0 (0)	1 (10)	
Very high	4 (8)	0 (0)	4 (40)	
Fat mass (kg) (≥6 years) ^28^	32.21±16.6	35.18±2.75	20.94±2.80	**0.014**
Low	2 (4)	1 (2.6)	1 (10)	
Normal	16 (32)	13 (34.2)	3 (30)	
High	13 (26)	10 (26.3)	3 (30)	
Very high	17 (34)	14 (36.8)	3 (30)	
Visceral mass (kg) (≥6 years) ^28^	6.59±5.9	6.06±0.4	8.61±4.15	0.254
Normal	40 (80)	32 (94.1)	8 (100)	
High	2 (4)	2 (5.9)	0 (0)	
**Medical history**				
Family history of CD				
No	29 (58)	23 (59)	6 (54.5)	0.79
Yes	21 (42)	16 (41)	5 (45.5)	
Age at diagnosis	22.28±13.55	24.34±13.84	15.18±10.01	0.26
Symptoms				
No	3 (6)	1 (2.6)	2 (18.2)	0.054
Yes	47 (94)	38 (97.4)	9 (81.8)	
Severity of symptoms				
Mild	17 (34)	12 (30.8)	5 (45.5)	0.32
Moderate	16 (32)	12 (30.8)	4 (36.4)	
Severe	17 (34)	15 (38.5)	2 (18.2)	
Related illness				
No	38 (76)	29 (74.4)	9 (81.8)	0.609
Yes	12 (24)	10 (25.6)	2 (18.2)	
Medications				
No	39 (78)	29 (74.4)	10 (90.9)	0.242
Yes	11 (22)	10 (25.6)	1 (9.1)	
**Laboratory values**				
Albumin (3.5-5.5 g/dl) ^24^	4.41±1.05	4.57±0.72	3.84±1.72	**0.02**
Low	7 (14)	2 (5.1)	5 (45.5)	
Normal	36 (72)	32 (82.1)	4 (36.4)	
High	7 (14)	5 (12.8)	2 (18.2)	
Iron (μmol/L (SI units) ^21^	31.83±10.49	30.80±9.84	35.50±12.35	0.40
Low	19 (38)	15 (38.5)	4 (36.4)	
Normal	20 (40)	14 (35.9)	6 (54.5)	
High	11 (22)	10 (25.6)	1 (9.1)	
vit. B12 (pmol/l) ^22^	329.52±110.8	323.26±18.7	351.72±26.29	0.10
Low	8 (16)	8 (20.5)	0 (0)	
Normal	42 (84)	31 (79.5)	11 (100)	
High	0 (0)	0 (0)	0 (0)	
Folate (ng/mL) ^23^	14.79±3.36	14.51±3.35	15.78±3.38	0.273
Low	0 (0)	0 (0)	0 (0)	
Normal	45 (90)	35 (89.7)	10 (90.9)	
High	3 (6)	2 (5.1)	1 (9.1)	
**Lifestyle**				
Smoking				0.78
No	39 (78)	31 (79.5)	8 (72.7)	
Yes	11 (22)	8 (20.5)	3 (27.3)	
Alcohol (per week)				
No	41 (82)	30 (78.9)	10 (90.9)	0.36
Yes	9 (18)	8 (21.1)	1 (9.1)	
Physical activity (per week)				
No	36 (72)	29 (74.4)	7 (63.6)	0.69
Yes	14 (28)	10 (25.6)	4 (36.4)	
<1 time per week	4 (28.6)	4 (40)	0 (0)	0.13
2-3 times per week	10 (71.4)	6 (60)	4 (100)	
>4 times per week	0 (0)	0 (0)	0 (0)	

^a^ Gluten free diet.

^b^ Weight change is calculated by subtracting weight after from the weight before and the significance is determined by paired sample t-test.

^c^ Subjects aged under 20 years were excluded from this analysis.

^d^ p≤0.05 indicates the significance of values of independent tests between the means of female and males.

As for the medical history, around half the study participants had a family history of CD. The mean age at diagnosis was 22.28±13.55 (range=1-57 years). Symptoms related to CD such as abdominal bloating, diarrhea, headache, and flatulence appeared in most participants (94%) and 6% were asymptomatic. Around 66% were facing moderate to severe symptoms. The participants were asked about comorbidities along with CD; only 24% of them had CD with other diseases of which dermatitis, arthritis, lactose intolerance and food allergy. As for the medications, 78% of the study participants reported being on medical treatments for CD symptoms.

As for the laboratory values, hypoalbuminemia was observed in 14% of the study population. Women were having significantly higher mean and SD levels of albumin compared to men (p-value=0.02). Out of 50 participants, 38% and 16% were presenting low serum levels of iron and vitamin B12, respectively. None of the study participants presented with folate deficiency. As for the lifestyle behaviors, the majority of participants were non-smokers, non-drinkers and physically inactive ( Table 2).

The food-related behaviors assessed among the study participants are presented in Table 3. Around 80% of the study participants reported reading nutrition labels when shopping groceries. Moreover, almost 96% of them were following a nutritional diet of which was the GFD. To assess their adherence to GFD, the examination of 96% of the participant’s three-day records reported low intake of gluten (<3g per day). Only 4% weren’t adhering to the GFD recommendations. As reported by the participants, some barriers including family ignorance (among 6% of the population), language on the nutrition labels (among 20% of the population) and expensive GF products (among 78% of the population) were limiting the adherence to GFD. It was observed that 72% of the study population had good cooking experience and 64% of the study participants were involved in preparing meals for themselves. On the other hand, almost all the subjects were unaware of a possible cross-contamination during cooking. Despite that, 82% of the subjects with CD preferred fast foods, although they consumed their meals frequently at home (92%). It was reported that the adherence to GFD by these participants induced a hunger feeling during the day. Most of our study population necessitated extra care by family members regarding cooking and eating. No significant differences were observed between genders.

**Table 3.** Food-related behaviors among the study population, overall and by gender. GF: gluten-free; GFD: gluten-free diet; F: female; M: male.

Outcome	Overall (N=50) N (%)	F (N=39;78%) N (%)	M (N=11; 22%) N (%)	p-value
Reading labels when eating and shopping				
No	10 (20)	6 (15.4)	4 (36.4)	0.12
Yes	40 (80)	33 (84.6)	7 (63.6)	
Following diet				
None	2 (4)	2 (5.2)	1 (5.22)	0.32
GF diet	48 (96)	37 (94.9)	10 (90.9)	
**Adherence to GFD** ^**a**^				0.06
No	1 (2)	0 (0)	1 (9.1)	
Yes	48 (96)	38 (100)	10 (90.9)	
Family support in GF diet’s adherence				0.63
No	3 (6)	3 (7.7)	0 (0)	
Yes	47 (94)	36 (92.3)	11 (100)	
Language of food label as barriers in GF diet’s adherence				0.70
No	40 (80)	32 (82.1)	8 (72.7)	
Yes	10 (20)	7 (17.9)	3 (27.3)	
Financial barriers towards GF diet’s adherence				0.84
No	11 (22)	9 (23.1)	2 (18.2)	
Yes	39 (78)	30 (76.9)	9 (81.8)	
Cooking experience				**0.026**
No	14 (28)	8 (20.5)	6 (54.5)	
Yes	36 (72)	31 (79.5)	5 (45.5)	
Self-preparation of meals				
No	18 (36)	12 (30.8)	6 (54.5)	0.147
Yes	32 (64)	27 (69.2)	5 (45.5)	
Awareness of cross-contamination during meal preparation				
No	49 (98)	38 (97.4)	11 (100)	0.592
Yes	1 (2)	1 (2.6)	0 (0)	
Fast foods vs home meals				
Fast foods	41 (82)	32 (82.1)	9 (81.8)	
Home meals	9 (18)	7 (17.9)	2 (18.2)	0.986
Eating out vs. eating at home				
Home	46 (92)	37 (94.9)	9 (81.8)	0.159
Eating out	4 (8)	2 (5.1)	2 (18.2)	
Feeling hungry most of the time				0.08
No	14 (30)	14 (35.9)	1 (9.1)	
Yes	25 (70)	25 (64.1)	10 (90.9)	
Looking for extra care in meals preparation				
No	10 (20)	8 (20.5)	2 (18.2)	0.847
Yes	40 (80)	31 (79.5)	9 (81.8)	

^a^ Patients presenting an ingestion of approximately 3–10 g of gluten per day were considered as non-adherent. ^12^^,^^25^

### Energy and protein intake compared to the recommended daily allowances (RDA)

Table 4 shows the mean energy and nutrients intake calculated through the three-day food records reported by the study participants or their families. Among female children aged four to eight years, the mean daily energy (E) and protein (P) intake were estimated as 1396 ±424.15 Kcal/d and 56.41 ±18.31 g/d, respectively. In comparison with the reference values of the recommended daily allowances (RDA), the daily intake of E and P was 130% and 268%, respectively. On the other hand, it was observed that female children aged 9-13 years had insufficient daily E intake (75% vs RDA) but surpassed by 317% the daily protein requirements. Similarly, among the female participant’s age groups 19-30, 31-50 and 50-70, there were inadequacies in the daily E intake along with overconsumption of protein per day. Otherwise, inadequacies in the daily E and P intake were observed among the male children (four to eight years) and those aged 19 to 30 years. Moreover, among those aged 9-13 and 31-50, the daily E intake was inadequate, but the P intake was high. No significant differences regarding the daily E and P intakes were observed between females and males, except in the 19-30 age group, where the daily energetic and protein ratios were high in females compared to males (p-value=0.024 and p=0.047, respectively).

**Table 4.** Energy and protein intake and their reference values according to recommended daily allowance (RDA) among the study population. EI: energy intake; PI: protein intake.

Variables	Overall	Female	RDA	% of adequacy	Male	RDA	% of adequacy	p-value (F vs M)
**4-8**								
EI (kCal)	1396±424.15	1564±88.6	1200	130	1060±483	1400	75	0.195
PI (g)	56.41±18.31	51.22±9.8	19	268	14.14±10	19	74	0.384
**9-13**								
EI (kCal)	1202.5± 272.2	1010±0.0	1600	75	1395±0.0	1800	77.5	- *
PI (g)	39.85±39.85	108.36	34	317	52.00	34	152	- *
**19-30**								
EI (kCal)	1564±177.26	2076.25±39.7	2200	94	1530± 255.2	2900	52.7	**0.024**
PI (g)	76.84±35.4	84.97± 8.86	46	184	92.7±12.92	56	60	**0.047**
**31-50**								
EI (kCal)	1758.3±772.4	1669.85±177.1	2200	76	2348±26.1	2400	98	0.942
PI (g)	178±35.43	71.99±9.702	46	156	84.73±11.5	56	151.8	0.423
**50-70**								
EI (kCal)	1701.08±296	1701.08±296	1900	89.5	- *	2300	-	- *
PI (g)	81.183±33	81.183±33	46	176	- *	56	-	- *

* The analysis couldn’t be generated due to the low sample size; the missing values in the table are due to the absence of males above the age of 50 years.

The average daily nutrient intake of calcium, iron and vitamin D is reported in Table 5. Among females and males, none of the study participants showed adherence to the daily recommendations for calcium and vitamin D intake. On the other hand, almost all the study participants from both genders showed a high daily consumption of iron.

**Table 5.** Average daily nutrient intake of subjects and percentage of adequacy according to recommended daily allowance (RDA).

Nutrient intake/Age	Mean of overall population	Female			Male			P-value (F vs M)
		Actual intake	RDA ^29^	% Adequacy	Actual intake	RDA ^29^	% Adequacy	
Calcium (mg/day) ^a^ ^,^ ^b^								
4-8	702.41±118.07	768.63±173.75	1000	77%	569.98±41.02	1000	57%	0.98
19-30	540.16±325.47	601.92±404.41	1000	60%	453.96±173.13	1000	45%	0.46
31-50	536.34±287.18	547.36±306.84	1000	55%	462.91±58.55	1000	46%	0.64
51-70	454.78±190.89	454.78±190.89	1200	38%	-	-	-	-
Iron (mg/day) ^a^ ^,^ ^b^								
4-8	16.303±3.11	22.48±7.48	10	194%	15.17±4.72	10	101%	0.09
19-30	30.80±5.55	33.49±6.39	18	158%	27.03±10.56	8	338%	0.59
31-50	17.22±3.95	17.58±4.49	18	98%	14.79±6.37	8	185%	0.81
51-70	18.81±7.56	18.81±7.56	8	235%	-	8	-	-
Vitamin D (ug/day) ^a^ ^,^ ^b^								
4-8	6.54±2.21	8.56±2.87	15	57%	2.52±0.06	15	17%	0.003
19-30	10.42±4.93	8.48±2.91	15	57%	13.12±11.82	15	88%	0.66
31-50	3.56±0.89	3.94±1.00	15	26%	1.00±0.39	15	7%	0.28
51-70	2.28±0.62	2.28±0.62	15	15%	-	-	-	-

^a^ In each age category, the age groups (13-14) were removed due to the limited number of patients.

^b^ The missing values in the table are due to the absence of males above the age of 50 years.

**Figure 1.**
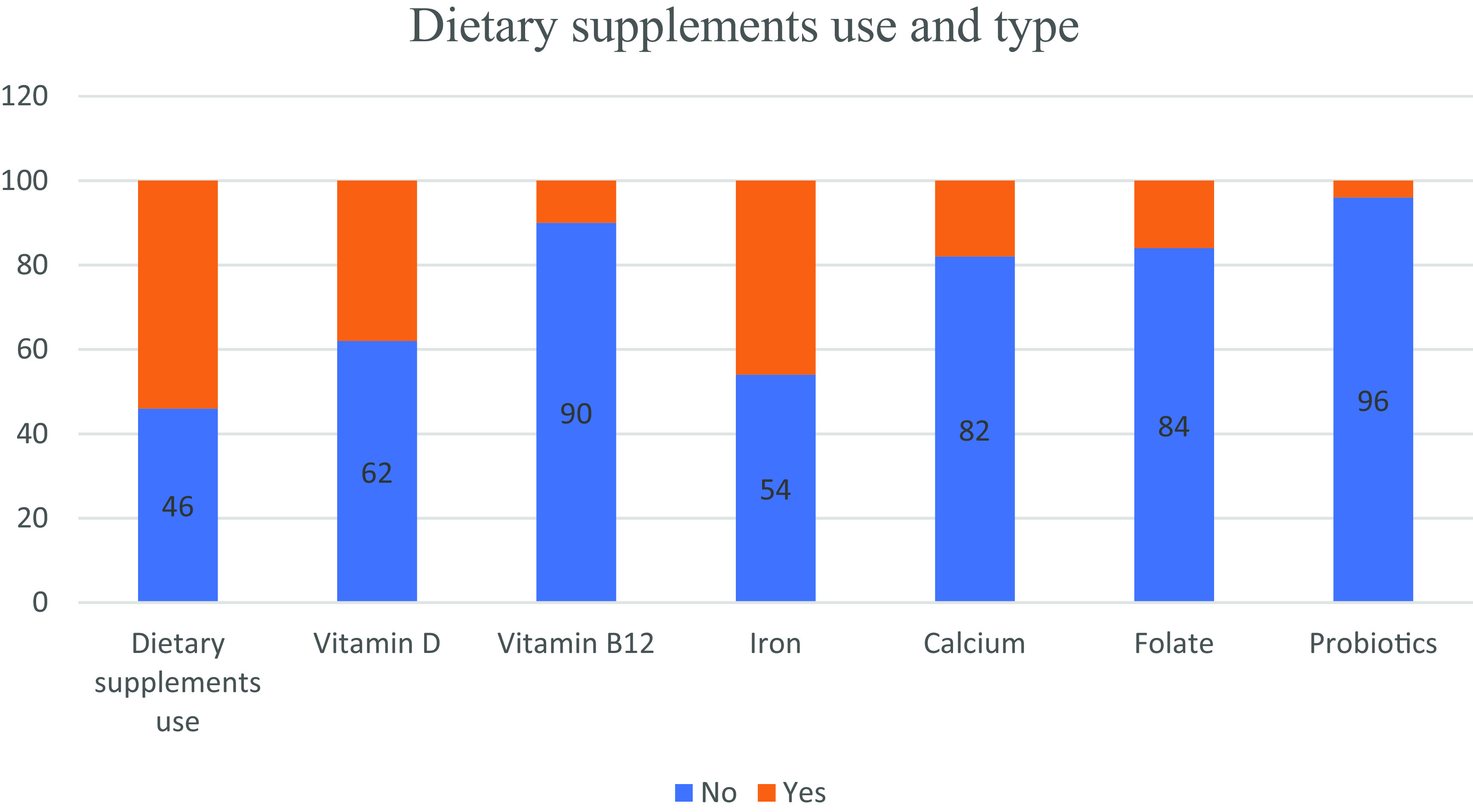
Dietary supplements use and type among study participants.

Dietary supplements were used by 54% of the study population of which 38%, 10%, 46%, 18%, 16% and 4% used vitamin D, vitamin B12, iron, calcium, folate and probiotics, respectively. No difference was observed between genders regarding DS use ( Table 6). According to Figure 1, it appears that the usage of DS among individuals with CD was not excessive.

**Table 6.** Supplements intake among study participants, by age group, by gender and reference to recommended daily allowance RDA for each element.

	Overall (N=50) N (%)	F (N=39;78%) N (%)	M (N=11; 22%) N (%)	P-value
Dietary supplements use				0.108
No	23 (46)	16 (41)	7 (63.6)	
Yes	27 (54)	23 (59)	4 (36.4)	
Vitamin D				0.125
No	31 (62)	22 (56.4)	9 (81.8)	
Yes	19 (38)	17 (43.6)	2 (18.2)	
Vitamin B12				0.909
No	45 (90)	35 (89.7)	10 (90.9)	
Yes	5 (10)	4 (10.3)	1 (9.09)	
Iron				0.158
No	27 (54)	19 (48.7)	8 (72.2)	
Yes	23 (46)	20 (51.3)	3 (27.8)	
Calcium				0.384
No	41 (82)	31 (79.4)	10 (90.9)	
Yes	9 (18)	8 (20.6)	1 (9.09)	
Folate				0.261
No	42 (84)	31 (81.5)	11 (100)	
Yes	7 (16)	7 (18.5)	0	
Probiotics				0.443
No	48 (96)	37 (94.8)	11 (22)	
Yes	2 (4)	2 (5.2)	0	

## Discussion

This study, the first of its kind in Lebanon, highlights the nutrition imbalances, P-E malnutrition, adherence to GFD, food-related behaviors and dietary supplements use among participants with CD. Our findings show that 78% of individuals with CD were women. This finding was higher than the data reported by Jansson-Knodell *et al.,* ^30^ and Ashtari *et al.* ^7^ which showed that the prevalence of women diagnosed with CD was 65% between 1990 and 2015 and the sero-prevalence of CD reported among women was 165 women out of 2000, respectively. On the other hand, our finding aligned the data reported by Singh *et al.* which showed that the prevalence of CD was higher among women compared to men. ^31^ In addition, according to Bai *et al.*, there are physiological differentials among genders in CD ^32^: “men have indirect evidence of greater malabsorption than females and have female-predominant associated diseases when they present with celiac disease”. ^32^ This study supported our finding concerning the remarked P-E deficiency among males compared to females in our study. Further, almost all the participants had symptoms related to CD such as abdominal bloating, diarrhea, headache, and flatulence; around 66% were facing moderate to severe symptoms and 78% of them reported being on a medical treatment for CD symptoms. These findings were two times higher than the prevalence reported by Stasi *et al.*, who showed that 22% of individuals with CD faced persistent abdominal symptoms. ^33^ In the present study, 14% presented hypoalbuminemia and mild to moderate malnutrition (10%-30% weight loss in the previous six months). This data agreed with Wierdsma *et al.* ^34^ where 17% of individuals with CD were malnourished (>10% undesired weight loss). On the other hand, the percentage of underweight women (5%) in our study wasn’t in accordance with Wierdsma *et al.* ^34^ in which 22% of the women were underweight and 29% of the patients were overweight (BMI > 25 kg/m ^2^). Our study showed that around 40% of individuals with CD had low muscle mass and 60% had high fat mass. This result was in contradiction with the results reported in a national study which showed that subjects with CD had a lower fat mass (17.8±2.0 kg) ^35^ but in concordance with two recent systematic reviews on children and adults that showed that after one year follow-up to GFD’ adherence, fat mass of CD patients significantly increased compared to that at baseline. ^36^^,^^37^ According to Agarwal *et al.*, patients with CD are more susceptible to develop metabolic syndromes and fatty liver diseases. ^38^ Thus, these patients should be regularly counseled by experienced dietitians for nutritional and metabolic factors about healthy diets and physical activity. However, the majority of participants in our study were physically inactive (72%). This prevalence of sedentary patterns among our study population was higher than that reported by Nestares *et al.* (37%) among children and adolescents ^39^ but lower than the prevalence of physical activity among women with CD in Poland (70%). ^40^

Out of 50 participants, 38% and 16% were presenting low serum levels of iron and vitamin B12, respectively. None of the study participants presented folate deficiency. These results showed consistency with a recent systematic review that showed high occurrence of hypoalbuminemia and anemia among individuals with CD. ^41^ Furthermore, micronutrient deficiencies are common features among individuals with CD. This was demonstrated in the study of Bledsoe *et al.* ^19^ where weight loss was seen in 25.2%, albumin was low in 19.7%, vitamin B _12_ was low in 5.3% and folate was low in 3.6% of cases. Moreover, it was shown that 87% of patients with CD in the study of Wierdsma, *et al.* ^34^ had low vitamin B _6_ (14.5%), folic acid (20%), and vitamin B _12_ (19%). Additionally, 46% of patients with CD had low serum iron, and 32% had anemia. ^34^

In our study, around 80% of participants read nutrition labels when shopping groceries. Reading and understanding food label’s topics have been discussed widely and have been associated with strict GFD adherence. ^42^ Our finding was in accordance with Butterworth *et al.*, who showed that amongst 74% of individuals with CD, some factors were in compliance with adherence to GFD: being a member of a celiac society, understanding food label’s language, and having access to gluten-free products with reasonable prices, regular dietetic and medical follow-up. ^43^ Causes of adherence/non-adherence to a GFD are multifactorial and differed according to their nature and magnitude. ^44^^–^^47^

Almost 96% of our population were following a GFD. To assess their adherence to GFD, the examination of the participant’s three-day records reported low intake of gluten (<3g per day) among 96% of the study participants. Only 4% weren’t adhering to the GFD recommendations. This finding was consistent with a study conducted in individuals with CD in Spain (92.5%). ^48^ According to a Spanish study, 72% of individuals with CD showed acceptable adherence which was associated with higher levels of self-efficacy. ^49^ Three studies from the UK showed that white patients were adhering to GFD in a range from 53 to 81%. ^50^ Non-adherence to GFD was also associated with diagnosis at younger ages and smoking. ^50^ This was not shown in our study. In Canada, GFD adherence was assessed and revealed that the percentage of strict adherence was only 56%. ^51^ The assessment of GFD adherence of 70 Swedish adolescents with CD showed that 86% of them were adherent to GFD five years after screening. ^52^ Moreover, of 5310 adult and adolescent Australians and New Zealanders with CD, 61% ashered to a GFD. ^53^ Three studies on adolescents from Brazil identified adherence rates from 36 to 86% taking into consideration that younger children (up to 12 years) were more likely to comply with the diet ^54^ while teenagers interrupted their diet purposefully. Parents of pediatric patients with CD from the Slovak Republic, reported a GFD adherence of 69% by children. ^55^ In addition, among 38 studies, the adherence was ranging from 42 and 91%. ^49^ According to a recent systematic review of 49 studies, the adherence rates were ranging from 23 to 98%. ^56^ This wide variability in adherence rates may be explained by the different populations examined ( *e.g.*, adults, adolescents, children, ethnic minorities), but also by the different tools used for evaluating adherence.

As reported by the participants, some barriers including family ignorance (among 6% of the population), language on the nutrition labels (among 20% of the population) and expensive GF products (among 78% of the population) were limiting the adherence to GFD. These findings were in concordance with Muhammad *et al.,* ^44^ who showed that not understanding food labelling was associated with lower GF dietary adherence scores. A higher proportion of individuals in the United Kingdom (UK) reported difficulties in acquiring knowledge about GF products (ranging between 5% and 76%) and understanding of food labels (4%-53%). ^57^ It was observed that 72% of the study population had good cooking experience and 64% of the study participants prepared their meals. On the other hand, almost all the subjects were unaware of a possible cross-contamination during cooking food. Despite 82% of the subjects with CD preferring fast foods, they frequently consumed their meals at home (92%). Beyond cooking meals at home and cross-contamination, our findings were consistent with the findings reported in a recent systematic review indicating that the most significant barriers limiting the adherence to GFD included: “lower knowledge of CD” (35%); “restaurant/supermarket shopping” (30%); “poor patient education from practitioner” (17.5%); and “low intention/motivation to adhere to a GFD (17.5%)”. ^45^

In our study, patients with CD had an inadequate total daily energy intake. This finding was concordant with several studies from Poland, Germany and Spain. ^58^^–^^60^ As for nutrients intake, among females and males, none of the study participants showed adequacy to the daily recommendations for calcium and vitamin D intake. These results, altogether, showed an unbalanced diet in terms of micronutrients, that were in accordance with those shown in several studies. ^14^^,^^61^^–^^63^ On the other hand, almost all the study participants from both genders showed a high daily consumption of iron. This finding contradicts the results reported in other studies. ^60^^,^^64^^–^^66^

DS were used by 54% of the study population of which 38%, 10%, 46%, 18%, 16% and 4% used vitamin D, vitamin B12, iron, calcium, folate and probiotics, respectively. It appears that the usage of DS among individuals with CD wasn’t in excess of that reported in the general population in Lebanon. ^67^ In our study, the most commonly used DS was vitamin D and the least used one was prebiotics. According to the literature, gastrointestinal symptoms can be alleviated through prebiotics intake. It has been shown that *Bifidobacterium infantis* can alleviate gastrointestinal symptoms if administered to CD patients before meals for three weeks. ^68^ This can explain the occurrence and persistence of gastrointestinal manifestations among the majority of participants in the current study.

### Strength and limitations

The crucial strength of this study is that it fulfills a comprehensive assessment of dietary intake and food-related patterns, biochemical parameters, body composition, and physical activity. However, this study had some limitations: 1) the selected group of patients doesn’t represent the general population with CD in Lebanon; 2) it was a cross-sectional study and, thereby, our findings must be interpreted with caution; 3), physical activity levels were self-reported; 4) there is an important limitation of data on caloric, macro- and micronutrient composition of the recalled three-day record. The low sample size must also be considered.

## Conclusion

Monitoring nutritional status using dietary, anthropometric, blood tests and assessing physical activity are the key components in the management of CD. The ideal GFD should be balanced with macronutrients and micronutrients, with reasonable access and prices. Fortification/enrichment of GF foods that are frequently consumed should be encouraged to avoid deficiencies. This underlines the critical role of dietitians in education and maintenance of healthy GFD among individuals with CD.

## Data availability

### Underlying data

Open Science Framework: Celiac disease_Nutrition_Lebanon, https://osf.io/qcfb3. ^69^

This project contains the following underlying data:

‐ SPSS Celiac.sav (raw questionnaire, morphometric and serology data)

### Extended data

Open Science Framework: Celiac disease_Nutrition_Lebanon, https://osf.io/qcfb3. ^69^

This project contains the following extended data:

‐ Questionnaire.pdf

Data are available under the terms of the Creative Commons Zero “No rights reserved” data waiver (CC0 1.0 Public domain dedication).

## References

[1] Therrien A Kelly CP Silvester JA : Celiac Disease: Extraintestinal Manifestations and Associated Conditions. *J. Clin. Gastroenterol.* 2020;54(1):8–21. 10.1097/MCG.0000000000001267) 31513026 PMC6895422

[2] Lebwohl B Rubio-Tapia A : Epidemiology, Presentation, and Diagnosis of Celiac Disease. *Gastroenterology.* 2021;160(1):63–75. 10.1053/j.gastro.2020.06.098 32950520

[3] Green P Cellier C : Celiac disease. *N. Engl. J. Med.* 2007;357(17):1731–1743. 10.1056/nejmra071600 17960014

[4] Al-Toma A Volta U Auricchio R : European Society for the Study of Celiac Disease (ESsCD) guideline for celiac disease and other gluten-related disorders. *United European Gastroenterol. J.* 2019;7(5):583–613. 10.1177/2050640619844125 31210940 PMC6545713

[5] Ludvigsson JF Ciacci C Green PHR : Outcome measures in celiac disease trials: The Tampere recommendations. *Gut.* 2018;67(8):1410–1424. 10.1136/gutjnl-2017-314853 29440464 PMC6204961

[6] Fedewa MV Bentley JL Higgins S : Celiac Disease and Bone Health in Children and Adolescents: A Systematic Review and Meta-Analysis. *J. Clin. Densitom.* 2020;23(2):200–211. 10.1016/j.jocd.2019.02.003 30833087

[7] Ashtari S Najafimehr H Pourhoseingholi MA : Prevalence of celiac disease in low and high risk population in Asia-Pacific region: a systematic review and meta-analysis. *Sci. Rep.* 2021;11(1):2383. 10.1038/s41598-021-82023-8 33504878 PMC7841177

[8] El-Metwally A Toivola P AlAhmary K : The Epidemiology of Celiac Disease in the General Population and High-Risk Groups in Arab Countries: A Systematic Review. *Biomed. Res. Int.* 2020;2020:6865913–6865917. 10.1155/2020/6865917 32596351 PMC7292982

[9] Barada K : Celiac disease in Middle Eastern and North African countries: A new burden?. *World J. Gastroenterol.* 2010;16(12):1449–1457. 10.3748/wjg.v16.i12.1449 20333784 PMC2846249

[10] Gładyś K Dardzińska J Guzek M : Celiac Dietary Adherence Test and Standardized Dietician Evaluation in Assessment of Adherence to a Gluten-Free Diet in Patients with Celiac Disease. *Nutrients.* 2020;12(8):2300. 10.3390/nu12082300 32751809 PMC7468751

[11] O’Mahony S Howdle PD Losowsky MS : Management of patients with non-responsive celiac disease. *Aliment. Pharmacol. Ther.* 1996;10(5):671–680. 10.1046/j.1365-2036.1996.66237000.x 8899074

[12] Ciacci C Cirillo M Cavallaro R : Long-term follow-up of celiac adults on gluten-free diet: Prevalence and correlates of intestinal damage. *Digestion.* 2002;66(3):178–185. 10.1159/000066757 12481164

[13] Nazareth S Lebwohl B Tennyson CA : Dietary Supplement Use in Patients with Celiac Disease in the United States. *J. Clin. Gastroenterol.* 2015;49(7):577–581. 10.1097/mcg.0000000000000218 25203364

[14] Taetzsch A Das SK Brown C : Are Gluten-Free Diets More Nutritious? An Evaluation of Self-Selected and Recommended Gluten-Free and Gluten-Containing Dietary Patterns. *Nutrients.* 2018;10(12):1881. 10.3390/nu10121881 30513876 PMC6317051

[15] Pelkowski TD Viera AJ : Celiac disease: diagnosis and management. *Am. Fam. Physician.* 2014;89(2):99–105. 24444577

[16] Cranney A Zarkadas M Graham ID : The Canadian Celiac Health Survey. *Dig. Dis. Sci.* 2007;52(4):1087–1095. 10.1007/s10620-006-9258-2 17318390

[17] Harmouche-Karaki M Mahfouz M Obeyd J : Development and validation of a quantitative food frequency questionnaire to assess dietary intake among Lebanese adults. *Nutr. J.* 2020;19(1):65. 10.1186/s12937-020-00581-5 32631430 PMC7339409

[18] Moghames P Hammami N Hwalla N : Validity and reliability of a food frequency questionnaire to estimate dietary intake among Lebanese children. *Nutr. J.* 2016;15:4. 10.1186/s12937-015-0121-1 26753989 PMC4709981

[19] Bledsoe AC King KS Larson JJ : Micronutrient Deficiencies Are Common in Contemporary Celiac Disease Despite Lack of Overt Malabsorption Symptoms. *Mayo Clin. Proc.* 2019;94(7):1253–1260. 10.1016/j.mayocp.2018.11.036 31248695

[20] Marteau P Verkarre V André C : Une Diarrhée avec carence en vitamine B12 [Diarrhea with vitamin B12 deficiency]. *Gastroenterologie clinique et biologique.* 2001;25(5):495–497. 11534515

[21] Pagana KD Pagana TJ Pagana TN : *Mosby's Diagnostic and Laboratory Test Reference.* Elsevier;2021.

[22] Abildgaard A Knudsen CS Hoejskov CS : Reference intervals for plasma vitamin B12 and plasma/serum methylmalonic acid in Danish children, adults and elderly. *Clinica Chimica Acta; International Journal of Clinical Chemistry.* 2022;525:62–68. 10.1016/j.cca.2021.12.015) 34942168

[23] Fishbach : *Manual of laboratory and disgnostic tests.* 9th ed. Philadephia, PA: Lippincott Williams & Wilkins;2015. Reference Source

[24] McPherson RA : Laboratory statistics. *Henry's Clinical Diagnosis and Management by Laboratory Methods.* 2011;109–118. 10.1016/b978-1-4377-0974-2.00009-9

[25] Kotze LM : Gynecologic and obstetric findings related to nutritional status and adherence to a gluten-free diet in Brazilian patients with celiac disease. *J. Clin. Gastroenterol.* 2004;38(7):567–574. 10.1097/01.mcg.0000131720.90598.6a) 15232359

[26] Braun V Clarke V : What can “thematic analysis” offer health and wellbeing researchers?. *Int. J. Qual. Stud. Health Well Being.* 2014;9(1):26152. 10.3402/qhw.v9.26152 25326092 PMC4201665

[27] DRI Activities Update:2020.Accessed April 28, 2022. Reference Source

[28] Frequently asked questions: Omron Healthcare.Accessed April 28, 2022. Reference Source

[29] Dietary reference intakes summary tables.Accessed April 28, 2022. Reference Source Reference Source

[30] Jansson-Knodell CL King KS Larson JJ : Gender-Based Differences in a Population-Based Cohort with Celiac Disease: More Alike than Unalike. *Dig. Dis. Sci.* 2018;63(1):184–192. 10.1007/s10620-017-4835-0 29127609 PMC5961510

[31] Singh P Arora A Strand TA : Global Prevalence of Celiac Disease: Systematic Review and Meta-analysis. *Clinical Gastroenterology and Hepatology: The Official Clinical Practice Journal of the American Gastroenterological Association.* 2018;16(6):823–836.e2. 10.1016/j.cgh.2017.06.037 29551598

[32] Bai D Brar P Holleran S : Effect of gender on the manifestations of celiac disease: evidence for greater malabsorption in men. *Scand. J. Gastroenterol.* 2005;40(2):183–187. 10.1080/00365520510011498 15764149

[33] Stasi E Marafini I Caruso R : Frequency and Cause of Persistent Symptoms in Celiac Disease Patients on a Long-term Gluten-free Diet. *J. Clin. Gastroenterol.* 2016;50(3):239–243. 10.1097/MCG.0000000000000392 26280705

[34] Wierdsma NJ van Bokhorst-de van der Schueren MA Berkenpas M : Vitamin and mineral deficiencies are highly prevalent in newly diagnosed celiac disease patients. *Nutrients.* 2013;5(10):3975–3992. 10.3390/nu510397517%) 24084055 PMC3820055

[35] Bassil M Hassan H : Compromised Nutritional Status of Adults with Celiac Disease on a Gluten Free Diet: The Case of Lebanon. *FASEB J.* 2017;31(1 Supplement):965–968.27920150

[36] Vereczkei Z Farkas N Hegyi P : It Is High Time for Personalized Dietary Counseling in Celiac Disease: A Systematic Review and Meta-Analysis on Body Composition. *Nutrients.* 2021;13(9):2947. 10.3390/nu13092947 34578835 PMC8466091

[37] Houttu N Kalliomäki M Grönlund MM : Body composition in children with chronic inflammatory diseases: A systematic review. *Clin. Nutr. (Edinburgh, Scotland).* 2020;39(9):2647–2662. 10.1016/j.clnu.2019.12.027 32035751

[38] Agarwal A Singh A Mehtab W : Patients with celiac disease are at high risk of developing metabolic syndrome and fatty liver. *Intest. Res.* 2021;19(1):106–114. 10.5217/ir.2019.00136 32312034 PMC7873403

[39] Nestares T Martín-Masot R Teresa C de : Influence of Mediterranean Diet Adherence and Physical Activity on Bone Health in Celiac Children on a Gluten-Free Diet. *Nutrients.* 2021;13(5):1636. 10.3390/nu13051636 34068001 PMC8152289

[40] Kujawowicz K Mirończuk-Chodakowska I Witkowska AM : Dietary Behavior and Risk of Orthorexia in Women with Celiac Disease. *Nutrients.* 2022;14(4):904. 10.3390/nu14040904 35215554 PMC8879910

[41] Balaban DV Dima A Jurcut C : Celiac crisis, a rare occurrence in adult celiac disease: A systematic review. *World J. Clin. Cases.* 2019;7(3):311–319. 10.12998/wjcc.v7.i3.311 30746372 PMC6369385

[42] Madden AM Riordan AM Knowles L : Outcomes in celiac disease: A qualitative exploration of patients' views on what they want to achieve when seeing a dietitian. *J. Hum. Nutr. Diet.* 2016;29(5):607–616. 10.1111/jhn.12378 27196120

[43] Butterworth JR Banfield LM Iqbal TH : Factors relating to compliance with a gluten-free diet in patients with celiac disease: comparison of white Caucasian and South Asian patients. *Clin. Nutr. (Edinburgh, Scotland).* 2004;23(5):1127–1134. 10.1016/j.clnu.2004.02.009 15380905

[44] Muhammad H Reeves S Jeanes YM : Identifying and improving adherence to the gluten-free diet in people with celiac disease. *Proc. Nutr. Soc.* 2019;78(3):418–425. 10.1017/s002966511800277x 30630540

[45] Abu-Janb N Jaana M : Facilitators and barriers to adherence to gluten-free diet among adults with celiac disease: A systematic review. *J. Hum. Nutr. Diet.* 2020;33(6):786–810. 10.1111/jhn.12754 32348008

[46] Leffler DA Edwards-George J Dennis M : Factors that influence adherence to a gluten-free diet in adults with celiac disease. *Dig. Dis. Sci.* 2007;53(6):1573–1581. 10.1007/s10620-007-0055-3 17990115 PMC3756800

[47] Villafuerte-Galvez J Vanga RR Dennis M : Factors governing long-term adherence to a gluten-free diet in adult patients with celiac disease. *Aliment. Pharmacol. Ther.* 2015;42(6):753–760. 10.1111/apt.13319 26206401

[48] Fernández Miaja M Díaz Martín JJ Jiménez Treviño S : Study of adherence to the gluten-free diet in celiac patients. *Anales de Pediatria.* 2021;94(6):377–384. 10.1016/j.anpede.2020.06.012 34090634

[49] Hall NJ Rubin G Charnock A : Systematic review: adherence to a gluten-free diet in adult patients with celiac disease. *Aliment. Pharmacol. Ther.* 2009;30(4):315–330. 10.1111/j.1365-2036.2009.04053.x 19485977

[50] Wieser H Ruiz-Carnicer Á Segura V : Challenges of monitoring the gluten-free diet adherence in the management and follow-up of patients with celiac disease. *Nutrients.* 2021;13(7):2274. 10.3390/nu13072274 34209138 PMC8308436

[51] Silvester JA Weiten D Graff LA : Living gluten-free: Adherence, knowledge, lifestyle adaptations and feelings towards a gluten-free diet. *J. Hum. Nutr. Diet.* 2015;29(3):374–382. 10.1111/jhn.12316 25891988

[52] Johansson K Norström F Nordyke K : Celiac dietary adherence test simplifies determining adherence to a gluten-free diet in Swedish adolescents. *J. Pediatr. Gastroenterol. Nutr.* 2019;69(5):575–580. 10.1097/mpg.0000000000002451 31335839

[53] Halmos EP Deng M Knowles SR : Food knowledge and psychological state predict adherence to a gluten-free diet in a survey of 5310 Australians and New Zealanders with celiac disease. *Aliment. Pharmacol. Ther.* 2018;48(1):78–86. 10.1111/apt.14791 29733115

[54] Rodrigues M Yonamine GH Fernandes Satiro CA : Rate and determinants of non-adherence to a gluten-free diet and nutritional status assessment in children and adolescents with celiac disease in a tertiary Brazilian referral center: A cross-sectional and retrospective study. *BMC Gastroenterol.* 2018;18(1). 10.1186/s12876-018-0740-z 29351811 PMC5775619

[55] Rimárová K Dorko E Diabelková J : Compliance with gluten-free diet in a selected group of celiac children in the Slovak Republic. *Cent. Eur. J. Public Health.* 2018;26(Supplement):S19–S24. 10.21101/cejph.a5369 30817868

[56] Myléus A Reilly NR Green PHR : Rate, risk factors, and outcomes of nonadherence in pediatric patients with celiac disease: A systematic review. *Clin. Gastroenterol. Hepatol.* 2020;18(3):562–573. 10.1016/j.cgh.2019.05.046 31173891

[57] Muhammad H Reeves S Ishaq S : Adherence to a Gluten Free Diet Is Associated with Receiving Gluten Free Foods on Prescription and Understanding Food Labelling. *Nutrients.* 2017;9(7):705. 10.3390/nu9070705 28684693 PMC5537820

[58] Kikut J Konecka N Szczuko M : Quantitative assessment of nutrition and nutritional status of patients with celiac disease aged 13–18. *Rocz. Panstw. Zakl. Hig.* 2019;70(4):359–367. 10.32394/rpzh.2019.0084 31960667

[59] Martin J Geisel T Maresch C : Inadequate nutrient intake in patients with celiac disease: results from a German dietary survey. *Digestion.* 2013;87(4):240–246. 10.1159/000348850 23751356

[60] Ballestero-Fernández C Varela-Moreiras G Úbeda N : Nutritional Status in Spanish Adults with Celiac Disease Following a Long-Term Gluten-Free Diet Is Similar to Non-Celiac. *Nutrients.* 2021;13(5):1626. 10.3390/nu13051626 34066195 PMC8151936

[61] Thompson T Dennis M Higgins LA : Gluten-free diet survey: Are Americans with celiac disease consuming recommended amounts of fibre, iron, calcium and grain foods?. *J. Hum. Nutr. Diet.* 2005;18(3):163–169. 10.1111/j.1365-277x.2005.00607.x 15882378

[62] Hopman EGD Cessie S le Blomberg BM von : Nutritional Management of the gluten-free diet in young people with celiac disease in the Netherlands. *J. Pediatr. Gastroenterol. Nutr.* 2006;43(1):102–108. 10.1097/01.mpg.0000228102.89454.eb 16819385

[63] Al-Raee MB El-Sakka MA Al-Wahaidi AA : In depth analysis of risk factors for celiac disease amongst children under 18 years old in the Gaza strip. A cross sectional study. *Nutr. J.* 2012;11:97. 10.1186/1475-2891-11-97 23164160 PMC3511227

[64] Scricciolo A Elli L Doneda L : Efficacy of a High-Iron Dietary Intervention in Women with Celiac Disease and Iron Deficiency without Anemia: A Clinical Trial. *Nutrients.* 2020;12(7):2122. 10.3390/nu12072122 32708973 PMC7400798

[65] Unalp-Arida A Liu R Ruhl CE : Nutrient intake differs among persons with celiac disease and gluten-related disorders in the United States. *Sci. Rep.* 2022;12(1):5566. 10.1038/s41598-022-09346-y 35368035 PMC8976850

[66] Nestares T Martín-Masot R Labella A : Is a Gluten-Free Diet Enough to Maintain Correct Micronutrients Status in Young Patients with Celiac Disease?. *Nutrients.* 2020;12(3):844. 10.3390/nu12030844 32245180 PMC7146183

[67] Mohsen H Yazbeck N Al-Jawaldeh A : Knowledge, Attitudes, and Practices Related to Dietary Supplementation, before and during the COVID-19 Pandemic: Findings from a Cross-Sectional Survey in the Lebanese Population. *Int. J. Environ. Res. Public Health.* 2021;18(16):8856. 10.3390/ijerph18168856 34444605 PMC8395050

[68] Smecuol E Hwang HJ Sugai E : Exploratory, randomized, double-blind, placebo-controlled study on the effects of Bifidobacterium Infantis Natren life start strain super strain in active celiac disease. *J. Clin. Gastroenterol.* 2013;47(2):139–147. 10.1097/mcg.0b013e31827759ac 23314670

[69] Hoteit M : Celiac disease_Nutrition_Lebanon. 2022, May 10. 10.17605/OSF.IO/QCFB3

