# Supplementary material for: Nutritional status, nutrient imbalances, food-related behaviors and dietary supplements use among patients with celiac disease on a gluten free diet in Lebanon: a national cross-sectional study
Source: F1000Res. 2023 Apr 18;11:725. Originally published 2022 Jul 1. [Version 3] doi: 10.12688/f1000research.121859.3 (PMC10119616; doi:10.12688/f1000research.121859.3)
Supplement: Peer review history

# Peer review: Reviewer response for version 3

**DOI:** 10.5256/f1000research.144320.r170059

**Published:** 2023-04-20

**Authors:** Hamed R. Takruri

**Affiliations:** 1 Department of Nutrition and Food Technology, Faculty of Agriculture, The University of Jordan, Amman, Amman Governorate, Jordan

Approved.

Is the work clearly and accurately presented and does it cite the current literature?

Yes

If applicable, is the statistical analysis and its interpretation appropriate?

Yes

Are all the source data underlying the results available to ensure full reproducibility?

Yes

Is the study design appropriate and is the work technically sound?

Partly

Are the conclusions drawn adequately supported by the results?

Partly

Are sufficient details of methods and analysis provided to allow replication by others?

Yes

Reviewer Expertise:

Human Nutrition and Diet Therapy

I confirm that I have read this submission and believe that I have an appropriate level of expertise to confirm that it is of an acceptable scientific standard.

# Peer review: Reviewer response for version 2

**DOI:** 10.5256/f1000research.140619.r156224

**Published:** 2022-12-05

**Authors:** Anil K. Verma, Yeliz Serin

**Affiliations:** 1 Celiac Disease Research Laboratory, Department of Pediatrics, Università Politecnica delle Marche, Ancona, Italy; 2 Department of Nutrition and Dietetics, Faculty of Health Science, Çukurova University, Turkey

***“Food-related behaviors and GFD dietary adherence*** *: "Across the three-day food records, patients presenting a consumption between 3 to 10 g of gluten per day were considered as non-adherent" In this line, please explain how you calculated gluten intake from the diet record? Which method did you use?" *

- *For dietary compliance, patients were accurately interviewed by a trained physician and dietitian to assess the habitual intake of serving of gluten-containing food per week (e.g. pasta, pizza, bread, or cookies). Compliance to gluten-free diet was defined as follows: very low = more than one serving of gluten containing food/week; low = up to one serving of gluten-containing food/week; good = no gluten-containing food/week. Then based on the three days record the g/d data of gluten was investigated. We followed the same methodologies adopted in the below 2 studies:*
   
  - *Ciacci, C., Cirillo, M., Cavallaro, R., & Mazzacca, G. (2002). Long-term follow-up of celiac adults on gluten-free diet: prevalence and correlates of intestinal damage. Digestion, 66(3), 178–185. https://doi.org/10.1159/000066757*
  - *Kotze L. M. (2004). Gynecologic and obstetric findings related to nutritional status and adherence to a gluten-free diet in Brazilian patients with celiac disease. Journal of clinical gastroenterology, 38(7), 567–574. https://doi.org/10.1097/01.mcg.0000131720.90598.6a”*

**(Comment quoted from the author response comment to Anil K. Verma and Yeliz Serin’s first report.)**

**Reviewers' comment:** 

 I think there is still confusion about how the authors calculate the amount of gluten.

 According to their explanations; they assessed the habitual intake of serving of gluten-containing food per week. I think they should give more comprehensive explanation. Because the per serving of gluten-containing food doesn’t equal to the gluten content per serving of food. Gluten is a protein that found in foods. And not every gluten-containing food has equal protein or gluten.

 How much gluten does gluten-containing food contain per serving? Does it differ from one food to another (for example, pizza, bread... etc.)?

 And how much gluten is necessary to be classified as you mentioned low, good etc.?

Is the work clearly and accurately presented and does it cite the current literature?

Partly

If applicable, is the statistical analysis and its interpretation appropriate?

I cannot comment. A qualified statistician is required.

Are all the source data underlying the results available to ensure full reproducibility?

Yes

Is the study design appropriate and is the work technically sound?

Partly

Are the conclusions drawn adequately supported by the results?

Yes

Are sufficient details of methods and analysis provided to allow replication by others?

Yes

Reviewer Expertise:

NA

We confirm that we have read this submission and believe that we have an appropriate level of expertise to confirm that it is of an acceptable scientific standard, however we have significant reservations, as outlined above.

# Response

**Published:** 2022-12-06

**Authors:** Maha Hoteit

**Affiliations:** Lebanese University, Lebanon

Dear Anil,

 Thank you again for raising this comment. We remarked that this sentence in the manuscript confused many readers. Thus, the authors decided to omit it and replace it by "adherence to recommendations with regards to gluten free diet" without talking about numbers and gluten content. 

 This was amended in the modified version of the manuscript.

 Regards

# Peer review: Reviewer response for version 1

**DOI:** 10.5256/f1000research.133767.r151836

**Published:** 2022-10-10

**Authors:** Anil K. Verma, Yeliz Serin

**Affiliations:** 1 Celiac Disease Research Laboratory, Department of Pediatrics, Università Politecnica delle Marche, Ancona, Italy; 2 Department of Nutrition and Dietetics, Faculty of Health Science, Çukurova University, Turkey

**Title:** Nutritional status, nutrient imbalances, food-related behaviors, and dietary supplements use among patients with celiac disease in Lebanon: a national cross-sectional study

**Opinion**

 In this manuscript, The authors have investigated the nutritional status, food-related behaviors, caloric, macro-, and micronutrient intake and dietary supplements use among individuals with CD in Lebanon. The authors claim, there is no data related to this issue in Lebanon. On a general search on the web, I did not find any related articles from Lebanon. Hence, I appreciate the authors for paying attention to the nutritional status of celiac patients living in Lebanon. However, I have multiple doubts that I am summarising below:

**Method **

- **In the Selection procedure part** *:* In the ‘exclusion criteria’ the authors mentioned that they eliminated patients with malnutrition. But in Table 2, they assessed the risk of malnutrition in the patients with malnutrition. Please clarify it.
- **The line of measurements**: " *Weight and height were measured using automated scale and stadiometer. Body composition including body fat, lean body mass, and visceral fat was assessed using Bio-impedancemetry body composition analyzer (BF-511, OMRON)"* Please explain the standardization of measurements.
- **Laboratory value** *: *The authors mentioned the collected data from three different centers. So, I think, the normal range of parameters could be changed. How did the authors standardized the laboratory results?
- **Food-related behaviors and GFD dietary adherence** *: "Across the three-day food records, patients presenting a consumption between 3 to 10 g of gluten per day were considered as non-adherent"* In this line, please explain how you calculated gluten intake from the diet record? Which method did you use?
- **Food record calculation:** The authors showed the energy and nutrient intake in Tables 4, and 5, but they don't explain how they calculated this parameter from the diet? Which program did they use for the calculation?
- **Statistical analysis** *: *The authors did not mention the normality of the data set. How did they decide the normality test like (t-Test) without checking normality?
- Please also mention the full form of the SPSS first then give its abbreviation in the brackets e.g., XXX (SPSS)

**Results: **

- In data with a statistically significant difference, please indicate under tables that: 1) Which test did you use and 2) The cut of point of significant P value (like p<0,05 or 0,001).

**Discussion: **

- The discussion part contains multiple repeats of the results. On the other hand, The authors haven't interpreted the result (Like How, and why ..). For example: What are the factors that influenced adherence to GFD in Lebanon? Please after comparing the results with other studies, also give original comments related to your data.
- Please first write the whole explanation of this term " *P-E malnutrition*" (Protein-energy malnutrition)
- *"In* *our study, the most commonly used DS was vitamin D and the least used one was prebiotics" *The authors mentioned prebiotics, I think it was written by mistake. Please clarify this term whether probiotic or prebiotic?
- Villous Atrophy takes place in the small bowel only, no need to specify its location small bowel. Alternatively, the authors may write, partial to total villous atrophy. (First line of the main introduction)
- The first line of the Selection procedure contains results (February to July 2018). Please shift this in the results section.

Is the work clearly and accurately presented and does it cite the current literature?

Partly

If applicable, is the statistical analysis and its interpretation appropriate?

I cannot comment. A qualified statistician is required.

Are all the source data underlying the results available to ensure full reproducibility?

Yes

Is the study design appropriate and is the work technically sound?

Partly

Are the conclusions drawn adequately supported by the results?

Yes

Are sufficient details of methods and analysis provided to allow replication by others?

Yes

Reviewer Expertise:

celiac disease, Gluten related disorders, HLA-DQ distribution, Non-celiac gluten sensitivity

We confirm that we have read this submission and believe that we have an appropriate level of expertise to confirm that it is of an acceptable scientific standard, however we have significant reservations, as outlined above.

# Response

**Published:** 2022-11-01

**Authors:** Maha Hoteit

**Affiliations:** Lebanese University, Lebanon

**Dear Reviewer 3, we modified the original manuscript according to your comments:**

**Method**

- ***"In the Selection procedure part**: In the ‘exclusion criteria’ the authors mentioned that they eliminated patients with malnutrition. But in Table 2, they assessed the risk of malnutrition in the patients with malnutrition. Please clarify it."*
  - All the patients collected in this study were referred to us by their physicians. According to their medical files, these patients weren’t malnourished. We excluded those who were diagnosed by physicians as malnourished patients because this interferes with our objectives. However, in our study, we were assessing the risk of malnutrition according to many specific criteria of which weight loss, albumin….
- ***"The line of measurements**: "Weight and height were measured using automated scale and stadiometer. Body composition including body fat, lean body mass, and visceral fat was assessed using Bio-impedancemetry body composition analyzer (BF-511, OMRON)" Please explain the standardization of measurements."*
   
  - Percentage body fat (%BF) was measured by whole-body BIA to the nearest 0.1% using a digital scale/body composition monitor (Omron BF 511, 50 kHz, 500 *µ*A, Kyoto, Japan), which includes an 8-sensor technology using both hands and feet. The participants stood with bare feet on electrodes on the scale with their knees and back straight while grasping a handle that also includes electrodes with both their hands horizontally raised, elbows extended straight, and maintaining a 90°-angle to the body. A previous study that compared body composition estimates using BIA devices with DXA and whole body magnetic resonance imaging indicated that the use of devices with additional hand electrodes provides a more accurate prediction of body composition and are suitable for public use. In order to determine percentage body fat, the device uses electrical impedance, along with the participant’s height, weight, age, and gender to generate results. The readings were obtained in duplicates and the average recorded. According to the manufacturer’s instructions, percentage body fat was measured two hours or more after breakfast. Omron Healthcare references for age and gender were used to classify the study participants into low, normal, high, and very high %BF.
  - We followed these articles: A. Bosy-Westphal, W. Later, B. Hitze et al., “Accuracy of Bioelectrical Impedance Consumer Devices for Measurement of Body Composition in Comparison to Whole Body Magnetic Resonance Imaging and Dual X-Ray Absorptiometry,” *Obesity Facts*, vol. 1, no. 6, pp. 319–324, 2008.
  - Healthcare Omron, *Omron Body Composition Monitor. Instruction Manual BF 511*, Omron Healthcare Cooperation Ltd., Kyoto, Japan, 2008.

 

- ***"Laboratory value**: The authors mentioned the collected data from three different centers. So, I think, the normal range of parameters could be changed. How did the authors standardized the laboratory results?"*
  - The three centers were using same equipment and no issues were presented in the laboratory results.
- ***"Food-related behaviors and GFD dietary adherence**: "Across the three-day food records, patients presenting a consumption between 3 to 10 g of gluten per day were considered as non-adherent" In this line, please explain how you calculated gluten intake from the diet record? Which method did you use?"*
   
  - For dietary compliance, patients were accurately interviewed by a trained physician and dietitian to assess the habitual intake of serving of gluten-containing food per week (e.g. pasta, pizza, bread, or cookies). Compliance to gluten-free diet was defined as follows: very low = more than one serving of gluten containing food/week; low = up to one serving of gluten-containing food/week; good = no gluten-containing food/week. Then based on the three days record the g/d data of gluten was investigated. We followed the same methodologies adopted in the below 2 studies:
     
    - Ciacci, C., Cirillo, M., Cavallaro, R., & Mazzacca, G. (2002). Long-term follow-up of celiac adults on gluten-free diet: prevalence and correlates of intestinal damage. *Digestion*, *66*(3), 178–185. https://doi.org/10.1159/000066757
    - Kotze L. M. (2004). Gynecologic and obstetric findings related to nutritional status and adherence to a gluten-free diet in Brazilian patients with celiac disease. *Journal of clinical gastroenterology*, *38*(7), 567–574. https://doi.org/10.1097/01.mcg.0000131720.90598.6a
- ***"Food record calculation:** The authors showed the energy and nutrient intake in Tables 4, and 5, but they don't explain how they calculated this parameter from the diet? Which program did they use for the calculation?"*
   
  - This was already available in text. The sentence is “ *In addition, three-day food records were collected and analyzed using Nutritionist Pro software (version 3.2, AXXYA) to assess caloric, micro- and macronutrients intake. The mean value and the standard deviation (SD) were used when interpreting the nutrient’s intake.*”
- ***"Statistical analysis**: The authors did not mention the normality of the data set. How did they decide the normality test like (t-Test) without checking normality?"*
   
  - **The normality was tested for sure.** The test used was Shapiro test.
- *"Please also mention the full form of the SPSS first then give its abbreviation in the brackets e.g., XXX (SPSS)"*
  - Done

**Results:**

- *"In data with a statistically significant difference, please indicate under tables that: 1) Which test did you use and 2) The cut of point of significant P value (like p<0,05 or 0,001)."*
  - Done

**Discussion:**

- *"The discussion part contains multiple repeats of the results. On the other hand, The authors haven't interpreted the result (Like How, and why ..). For example: What are the factors that influenced adherence to GFD in Lebanon? Please after comparing the results with other studies, also give original comments related to your data."*
  - Done
- *"Please first write the whole explanation of this term ****"P-E malnutrition" (Protein-energy malnutrition)"*
  - Done
- ""In our study, the most commonly used DS was vitamin D and the least used one was prebiotics" The authors mentioned prebiotics, I think it was written by mistake. Please clarify this term whether probiotic or prebiotic?"
  - *Probiotics*
- *"Villous Atrophy takes place in the small bowel only, no need to specify its location small bowel. Alternatively, the authors may write, partial to total villous atrophy. (First line of the main introduction)"*
  - Done
- *"The first line of the Selection procedure contains results (February to July 2018). Please shift this in the results section."*
  - Done

# Peer review: Reviewer response for version 1

**DOI:** 10.5256/f1000research.133767.r151835

**Published:** 2022-10-05

**Authors:** Hamed R. Takruri

**Affiliations:** 1 Department of Nutrition and Food Technology, Faculty of Agriculture, The University of Jordan, Amman, Amman Governorate, Jordan

Generally, the paper is good and informative, although there are some writing and structural mistakes; these are highlighted and commented on in the attached PDF article. Some long sentences should be rewritten and separated into short sentences. 

- It is not clear how the intake of gluten was estimated.
- It is unclear how the weight loss was evaluated; is it in reference to ideal body weight or to initial weight at the beginning of the study? 

The paper can be indexed after considering the notes mentioned above. 

 Please find further comments in the annotated pdf hyperlinked above or at the following link: *https://f1000researchdata.s3.amazonaws.com/linked/455693.Maha_Hoteit_article_121859_-_with_referees%27_%28Hamed_Takruri%29_notes.pdf*

Is the work clearly and accurately presented and does it cite the current literature?

Yes

If applicable, is the statistical analysis and its interpretation appropriate?

Yes

Are all the source data underlying the results available to ensure full reproducibility?

Yes

Is the study design appropriate and is the work technically sound?

Partly

Are the conclusions drawn adequately supported by the results?

Partly

Are sufficient details of methods and analysis provided to allow replication by others?

Yes

Reviewer Expertise:

Human Nutrition

I confirm that I have read this submission and believe that I have an appropriate level of expertise to confirm that it is of an acceptable scientific standard, however I have significant reservations, as outlined above.

# Response

**Published:** 2022-11-01

**Authors:** Maha Hoteit

**Affiliations:** Lebanese University, Lebanon

Dear Reviewer 2

 Thank you for your comments. We took into consideration all the raised comments and are available in the modified version. We provide below a reply point by point to your comments:

*"Generally, the paper is good and informative, although there are some writing and structural mistakes; these are highlighted and commented on in the attached PDF article. Some long sentences should be rewritten and separated into short sentences." *

- *"It is not clear how the intake of gluten was estimated*:"
  - For dietary compliance, patients were accurately interviewed by a trained physician and dietitian to assess the habitual intake of serving of gluten-containing food per week (e.g. pasta, pizza, bread, or cookies). Compliance to gluten-free diet was defined as follows: very low = more than one serving of gluten containing food/week; low = up to one serving of gluten-containing food/week; good = no gluten-containing food/week. Then based on the three days record the g/d data of gluten was investigated. We followed the same methodologies adopted in the below 2 studies:
    - Ciacci, C., Cirillo, M., Cavallaro, R., & Mazzacca, G. (2002). Long-term follow-up of celiac adults on gluten-free diet: prevalence and correlates of intestinal damage. *Digestion*, *66*(3), 178–185. https://doi.org/10.1159/000066757
    - Kotze L. M. (2004). Gynecologic and obstetric findings related to nutritional status and adherence to a gluten-free diet in Brazilian patients with celiac disease. *Journal of clinical gastroenterology*, *38*(7), 567–574. https://doi.org/10.1097/01.mcg.0000131720.90598.6a
- *"It is unclear how the weight loss was evaluated; is it in reference to ideal body weight or to initial weight at the beginning of the study?" *
  - In the last 6 months prior to the interview. We followed this study : Collins N. (2003). Protein-energy malnutrition and involuntary weight loss: nutritional and pharmacological strategies to enhance wound healing. *Expert opinion on pharmacotherapy*, *4*(7), 1121–1140. https://doi.org/10.1517/14656566.4.7.1121

*"The paper can be indexed after considering the notes mentioned above.*

*Please find further comments in the annotated pdf hyperlinked above or at the following link:* *https://f1000researchdata.s3.amazonaws.com/linked/455693.Maha_Hoteit_article_121859_-_with_referees%27_%28Hamed_Takruri%29_notes.pdf"*

  All the comments were taken into consideration.

# Peer review: Reviewer response for version 1

**DOI:** 10.5256/f1000research.133767.r143011

**Published:** 2022-07-22

**Authors:** Sherine Khater

**Affiliations:** 1 Department of Gastroenterology, AP-HP, Hôpital Européen Georges Pompidou, Paris, France

It is a very interesting study about nutritional status in CD (celiac disease) patients on GFD (gluten-free diets) in Lebanon, with many parameters studied. However, I have 2 remarks:  

1. Inclusion criteria include celiac disease patients attempting a GFD for at least 6 months. This should appear in the title of the article (the population is not CD patients at diagnosis but CD patients on GFD or attempting a GFD). This point should also appear in the abstract (Background section and Methods section). A very positive point was to evaluate the adherence to GFD of the patients included, which was high and thus reducing bias.  
  In the discussion part, some references are inadequate because they studied micronutrient deficiencies in CD patients at diagnosis, and not in CD patients following a GFD (example: references  32, 34, 41). These references should be replaced by others, for example: Cardo A et al, Nutritional Imbalances in Adult Celiac Patients Following a Gluten-Free Diet, Nutrients 2021, 13, 2877.
2. Concerning use of probiotics in CD, there is no strong evidence to date. For this reason, I think it is better to remove this section in the discussion: *"According to the literature, gastrointestinal symptoms can be alleviated through prebiotics intake. It has been shown that Bifidobacterium infantis can alleviate gastrointestinal symptoms if administered to CD patients before meals for three weeks. This can explain the occurrence and persistence of gastrointestinal manifestations among the majority of participants in the current study"*. Persistence of symptoms might more probably be explained by associated irritable bowel syndrome, or by inadvertent gluten ingestion.

Is the work clearly and accurately presented and does it cite the current literature?

Yes

If applicable, is the statistical analysis and its interpretation appropriate?

I cannot comment. A qualified statistician is required.

Are all the source data underlying the results available to ensure full reproducibility?

Yes

Is the study design appropriate and is the work technically sound?

Yes

Are the conclusions drawn adequately supported by the results?

Yes

Are sufficient details of methods and analysis provided to allow replication by others?

Yes

Reviewer Expertise:

Gastroenterology, celiac disease and malignant complications of celiac disease.

I confirm that I have read this submission and believe that I have an appropriate level of expertise to confirm that it is of an acceptable scientific standard, however I have significant reservations, as outlined above.

## References

[1] : Nutritional Imbalances in Adult Celiac Patients Following a Gluten-Free Diet. *Nutrients* .2021;13(8) : 10.3390/nu13082877 10.3390/nu13082877 34445038PMC8398893

# Response

**Published:** 2022-07-29

**Authors:** Maha Hoteit

**Affiliations:** Lebanese University, Lebanon

The authors would like to thank Reviewer 1 for reviewing the manuscript.

 All comments were taken into consideration.

 Changes in the manuscript are highlighted in track changes.

# Response

**Published:** 2022-11-01

**Authors:** Maha Hoteit

**Affiliations:** Lebanese University, Lebanon

Dear Reviewer 1

 All the comments raised are edited in the amended version.

 Many thanks
